# Insights into early pig domestication provided by ancient DNA analysis

**DOI:** 10.1038/srep44550

**Published:** 2017-03-16

**Authors:** Amke Caliebe, Almut Nebel, Cheryl Makarewicz, Michael Krawczak, Ben Krause-Kyora

**Affiliations:** 1 Institute of Medical Informatics and Statistics, Kiel University, 24105 Kiel, Germany; 2 Institute of Clinical Molecular Biology, Kiel University, 24105 Kiel, Germany; 3 Institute of Prehistoric and Protohistoric Archaeology, Kiel University, 24098 Kiel, Germany; 4 Max Planck Institute for the Science of Human History, 07745 Jena, Germany

## Abstract

Pigs (*Sus scrofa*) were first domesticated between 8,500 and 8,000 cal BC in the Near East, from where they were subsequently brought into Europe by agriculturalists. Soon after the arrival of the first domestic pigs in northern Europe (~4500 BC), farmers are thought to have started to incorporate local wild boars into their swine herds. This husbandry strategy ultimately resulted in the domestication of European wild boars. Here, we set out to provide a more precise geographic and temporal framework of the early management of suid populations in northern Europe, drawing upon mitochondrial DNA haplotype data from 116 Neolithic *Sus* specimens. We developed a quantitative mathematical model tracing the haplotypes of the domestic pigs back to their most likely geographic origin. Our modelling results suggest that, between 5000 and 4000 BC, almost all matrilines in the north originated from domesticated animals from the south of central Europe. In the following period (4000–3000 BC), an estimated 78–100% of domesticates in the north were of northern matrilineal origin, largely from local wild boars. These findings point towards a dramatic change in suid management strategies taking place throughout south-central and northern Europe after 4000 BC.

Pigs were first domesticated in the Near East around 8500 BC and subsequently brought into Europe by agriculturalists^1^. Ancient mitochondrial DNA (mtDNA) studies further indicate that, by 4500 BC, domesticated pigs bearing Near Eastern haplotypes appeared in northern Europe^2^. Soon thereafter, agriculturalists are thought to have incorporated local wild boars into their domesticated swine herds^1^,^2^. On the basis of a relatively small and geographically diffuse set of mtDNA data, it has been hypothesized that the Near Eastern mtDNA lineages were rapidly replaced by European haplotypes, apparently within 500 years or less, after the first introduction of Near Eastern domesticates into the region^1^.

Here, we set out to derive a more precise geographic and temporal framework for the early management of indigenous wild boar populations in northern Europe, a process which ultimately led to domesticated European lineages. We examined ancient mtDNA haplotype data from a large sample of 87 domestic and 29 wild Neolithic *Sus* specimens (Fig. 1). The study targeted an 80-bp highly informative fragment of the mitochondrial control region that provides ample information about maternal ancestry^1^,^3^,^4^,^5^. The presence of Near Eastern haplotypes Y1 and Y2 in pigs located in Europe has been previously interpreted as a marker of their domesticated status^1^, demonstrating the role of humans in facilitating the translocation of suids from the Near East into Europe. Reflecting the difficulty in resolving the domesticated status of animals by lineage alone in regions inhabited by both domesticates and their wild progenitors, *Sus* with the European haplotype A or C could represent either wild or domestic animals^2^. In order to better resolve the source and timing of pig management processes, we developed a stochastic model to trace the mtDNA haplotypes from nascent domestic pig populations of northern Europe of 5000 BC to 3000 BC back to their likely origin. Our study is the first to use such a quantitative model to ascertain the origin of prehistoric mtDNA haplotypes of northern domestic pigs.

## Results

### Study design and aim

In our sample of 116 Neolithic *Sus* specimens, we observed the four known mtDNA haplotypes Y1, Y2, A and C^1^ (Tables 1 and 2). Interestingly, Near Eastern haplotype Y1 appeared as late as 4000 to 3000 BC in northern domestic pigs.

**Figure 1:**
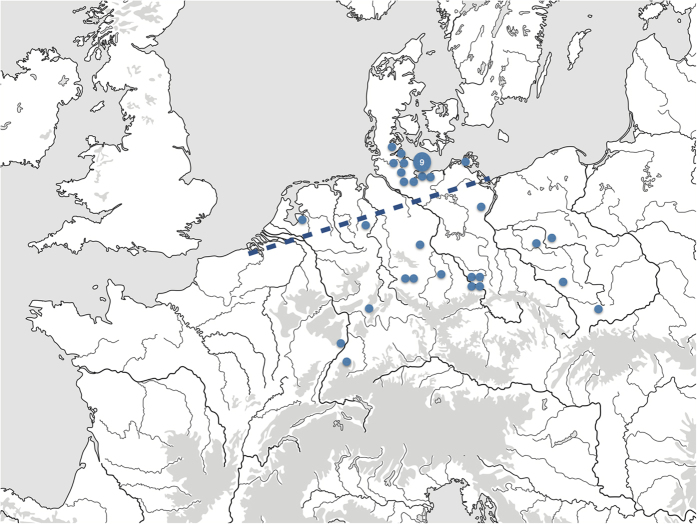
Location of archaeological *Sus* samples. The large circle represents nine geographically close archaeological sites. The dashed line separates the northern and the southern group of animals.

For the present study, pig remains recovered from sites located in the northern parts of Germany and the Netherlands were classified as belonging to the ‘northern group’ (n = 73), irrespective of haplotype (Fig. 1, Table S1). The northern region was home to late Mesolithic hunter-gatherers who eventually turned towards the use of domesticated plants and animals during the Neolithic (ca 4100 BC). Haplotype frequencies obtained from northern group pigs were compared to those among specimens recovered from southern Poland and central/southern Germany (‘southern group’, n = 43; Fig. 1), a region where Neolithic farmers had been established since ~5500 BC. The wild or domestic status of each specimen was determined based upon *(i)* its mtDNA haplotype (with Y1 or Y2 indicating domestic status) and *(ii)* standard metrical and non-metrical analyses (cp. Methods).

Animals were grouped further according to the time period (t_1_: 5500–5000 BC, t_2_: 5000–4000 BC, t_3_: 4000–3000 BC) and region (north *vs.* south) they had been living in (Tables 1 and 2). The three time periods and the two geographical regions were characterized by distinct subsistence strategies, landscape interactions and cultural material used. The first time period (t_1_) was largely defined by the presence of Mesolithic hunter-gatherer-fishers in the north and the first farmers in the south (5500–5000 BC). The second period (t_2_) (5000–4000 cal BC) was described in the north by the transition to early farmers and in the south by the super-regional Linienbandkeramik culture. The third period (t_3_) (4000–3000 cal BC) was defined by the emergence of more regional agriculturalist Funnel Beaker and Globular Amphora culture. The mtDNA haplotypes of the t_2_ sample of wild boars from the north (n = 29) were used as a reference for the haplotype distribution of wild boars in periods t_1_ and t_2_ (Table 2). In our stochastic model, we investigated time periods t_2_ and t_3_ separately. We determined the probability that the mtDNA of a northern domestic pig derived from an animal of certain status (domestic or wild) living in a certain region (north or south) during the previous time period (i.e., t_1_ or t_2_). Based on the occurrence of European haplotypes A and C in southern domestic pigs, it is clear that wild boars had been continuously integrated into the domestic livestock in the south^1^,^2^. Therefore, the group of southern domestic pigs consists not only of domestic pigs originally descended from domestic pigs from the Near East, but also of previously domesticated southern wild boars.

### Origin of mtDNA haplotypes of northern domestic pigs living 5000–4000 BC (t_2_)

**Figure 2:**
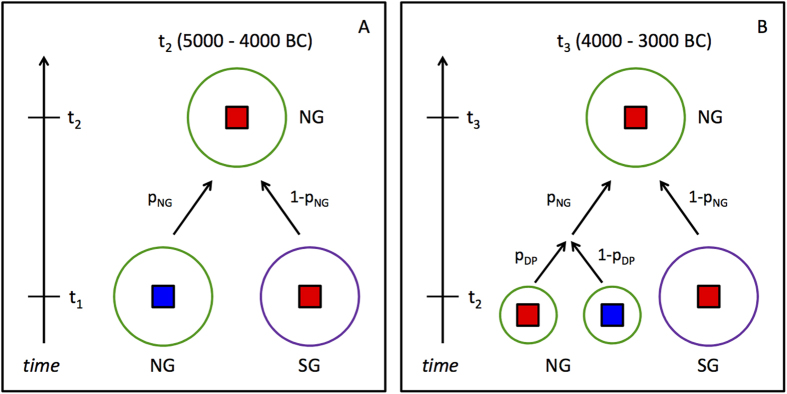
Stochastic models of the origin of mtDNA haplotypes in northern domestic pigs. NG, northern group (green); SG, southern group (purple); red square, domestic pig; blue square, wild boar; p_NG_, probability that a northern domestic pig descended from a northern pig of the previous time period; p_DP_, probability that a northern predecessor was domestic; (**A**): period 5000–4000 BC (t_2_) (**B**): period 4000–3000 BC (t_3_).

For time period t_1_ (5500–5000 BC), no domestic pigs have so far been identified in northern European contexts^6^,^7^. Therefore, the mtDNA of a northern t_2_ domestic pig can only originate from either a southern t_1_ domestic pig or a northern t_1_ wild boar (Fig. 2A). A single parameter is sufficient to describe this model, namely the probability p_NG_ that the mtDNA of a northern t_2_ domestic pig derived from a northern t_1_ wild boar. Our data yielded a maximum likelihood estimate of 0.20 for p_NG_ for two haplotypes (combining A and C on the one hand, and Y1 and Y2 on the other) and of 0 when all four haplotypes were considered. The 95% confidence interval for p_NG_ was [0, 0.83] for two haplotypes and [0, 0.28] for four haplotypes (Table 3, Figures S1A and B). The two-haplotype confidence interval is considerably larger because the European haplotypes A and C were combined, which masks the fact that in southern t_1_ domestic pigs, haplotype A is more frequent than haplotype C (A: 64% vs. C: 36% among the European haplotypes) whereas in northern t_1_ wild boars haplotype C is almost five times more frequent than A (A: 17% vs. C:83%). The four haplotype confidence interval takes the separate information about A and C into account and therefore yields a more precise estimate.

### Origin of mtDNA haplotypes of northern domestic pigs living 4000–3000 BC (t_3_)

**Figure 3:**
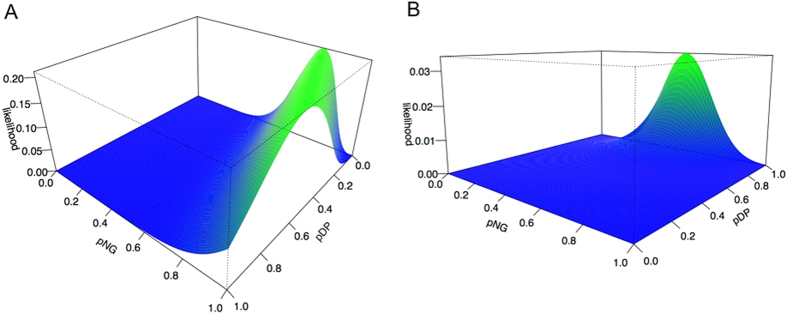
Likelihood surfaces for parameters p_NG_ and p_DP_ in time period t_3_ (4000–3000 BC). p_NG_, probability that a northern t_3_ domestic pig descended from a northern t_2_ pig; p_DP,_ probability that a northern predecessor (from time period t_2_) was domestic; for the definition of the likelihood function, see Methods, section Parameter estimation, Equation (3); (**A**): likelihood surface for two haplotypes (A and C *vs* Y1 andY2) (**B**): likelihood surface for four haplotypes (A, C, Y1, Y2)

Since domestic pigs were already present in northern Europe in time period t_2_, mtDNA haplotypes of northern t_3_ domestic pigs have three possible origins, namely southern t_2_ domestic pigs, northern t_2_ domestic pigs or northern t_2_ wild boars (Fig. 2B). In addition to parameter p_NG_, i.e. the probability that the haplotype was derived from a northern rather than a southern t_2_ animal, a second parameter p_DP_ is required to distinguish between northern domestic and northern wild predecessors (p_DP_ equals the probability that a northern predecessor was domestic rather than wild). For two haplotypes, maximum likelihood estimation did not yield a unique solution in our data. Instead, possible values of p_NG_ and p_DP_ were found to lie on a straight line (Fig. 3A). Consequently, p_NG_ was estimated to lie between 0.78 and 1 whereas p_DP_ was estimated to lie between 0 and 0.57 (Table 3). When all four haplotypes were considered, unique maximum likelihood estimates were obtained as 1 for p_NG_ and 0.57 for p_DP_ (Table 3, Fig. 3B).

## Discussion

Our results ensued from 116 Neolithic specimens, a relatively large number for an ancient DNA study, thus enabling us to perform sensible statistical modelling. To the best of our knowledge, our study has been the first to develop a quantitative model to assess the prehistoric origin of mtDNA haplotypes of northern domestic pigs. However, for precise estimation of the model parameters, the study sample size may have been only moderately sufficient, as is evidenced by the relatively large confidence intervals obtained. Nevertheless, a clear trend is apparent. For early period t_2_, our analysis suggests none or only little influx of wild boar from the north. Thus, whereas predominant maternal descent from southern domestic pigs is likely, it cannot be excluded that a minor proportion of pigs received their mtDNA from animals from the northern region. The confidence interval for p_NG_ (the probability that the mtDNA of a northern t_2_ domestic pig derived from a northern t_1_ wild boar) was smaller in the four-haplotype model, not least owing to the larger amount of information that was taken into account, so that the parameter estimates of the four-haplotype model can be assumed to be more accurate than those of the two-haplotype model. Qualitatively, however, the two- and four-haplotype models agreed well for both time periods, thereby increasing confidence in our results.

Previous zooarchaeological research suggests that domesticated pigs were first exploited in northern Europe at around 4100 BC^8^. According to a recent aDNA study, however, domesticated Near Eastern lineage animals may have been present in the region several centuries earlier, a result derived from a directly radiocarbon-dated specimen with haplotype Y1 (two sigma range: BC 4720–4582)^2^. The mtDNA results presented here further suggest that, during the early time period t_2_ (5000–4000 BC), female domestic pigs were introduced almost exclusively from the south. In the following time period t_3_ (4000–3000 BC), by contrast, imports from the south contributed little to the northern mtDNA genetic pool and, consequently, the mtDNA of northern European lineage domestic pigs should have originated mainly from local animals, a substantial proportion (between 43% and 100%) of which were wild boars. Our model highlights a clear shift in suid management strategies after 4000 BC, a pattern in agreement with the model generated by Larson *et al*.^1^, which drew from a much smaller and geographically more widespread sample. Based on the occurrence of Near Eastern haplotype Y1 as late as t_3_ (between 4000 and 3000 BC; Tables 1 and 2) it seems that, at least in the north, the replacement of Near Eastern mtDNA haplotypes by European ones could have taken longer (>500 years) than previously suggested^1^. Equally intriguing, our study revealed a high level of interbreeding between the early domestic northern populations and local wild boars. Notably, significant post-domestication gene flow from wild animals into managed swineherds is further corroborated by a recent genomic study on modern pigs^9^.

Why did early agriculturalists in northern Europe change their suid management strategy after 4000 BC? It may be that the availability of domesticated Near Eastern pigs in the earliest Neolithic settlements located in northern Europe was initially limited, reflecting their location at the end of the Neolithic ‘supply chain’. The reliability of Near Eastern-derived livestock was perhaps also unstable, particularly if pig husbandry strategies applied to Near Eastern domesticates initially failed to adjust for northern European environments and dietary conditions. The overall number of domestic pigs carried to the north by the first Neolithic agriculturalists along with their livestock may have been relatively small. As a case in point, domesticated sheep and goats, core constituents of the Near Eastern Neolithic livestock package, were also exploited at low intensities only in the earliest Neolithic settlements of the southwestern Baltic region, with their remains generally comprising no more than 10% of the faunal assemblage^10^,^11^ [Makarewicz, unpublished data]. A small domestic source population would have been susceptible to isolation, disease and over-harvesting which would have made it difficult to maintain a stable and viable swine herd. In addition, the material cultural record indicates that the contact between northern and southern European cultures was only sporadic at the time^8^,^12^. If this was the case, then the strategy of interbreeding locally abundant female wild boars with domestic males would have facilitated an increase in swine herd size without having to rely upon a limited and uncertain supply of Near Eastern domesticated pigs from the south. Moreover, local stock are usually well adapted to their specific environmental conditions, for instance, by showing greater resistance to endemic pathogens^13^. Out-crossing therefore may have produced more resilient, fertile and larger offspring^12^,^13^. Another explanation for the dominance of local European mtDNA genomes in later pig specimens could be that the corresponding genomes conferred a selective advantage in the north, for example, in terms of energy metabolism that would eventually lead to their fixation in European domesticates.

It has been suggested before that interbreeding between pigs and wild boars was mainly unintentional and resulted from chance encounters due to escaped feral domesticates, loose swine management systems and mobile swine herding^9^,^12^. Loose pig husbandry is thought to have been characteristic of European agricultural systems for millennia^14^. However, this scenario would not explain the introgression of European wild sow mtDNA haplotypes (i.e. of female animals) into expanding Neolithic swineherds; mating between domesticated female sows and European wild boars would not have changed the mtDNA pool of domestic herds. The latter instead would have required the incorporation of additional wild females, which could, for instance, be achieved by capture of wild female piglets as part of an active herd-building strategy. Therefore, a combination of loose management and intentional integration of female wild boars seems to have occurred.

The results of our quantitative analyses strongly suggest that pig management was an ever-evolving process which depended heavily upon interbreeding domesticated animals with local wild boar, in particular wild sows. This insight demonstrates how a change in animal handling ~6000 years ago may have influenced livestock composition up to the present day. What is more, our mathematical model presented here can also be adapted to other proxies (i.e. animals) and haploid DNA markers as well as scenarios and periods.

## Methods

### Sample specification and determination of the wild or domestic status of samples

The domestication status of the sampled *Sus* remains was determined in a two-stage process. Since Near Eastern mtDNA haplotypes Y1 and Y2 classify a pig as domestic with very high probability, samples with one of these two haplotypes were assigned domestic status (n = 25). Since pigs with European haplotype A or C can be either domestic or wild, we had to rely upon standard metrical and non-metrical morphological analyses for the classification of *Sus* with these haplotypes (n = 91).

Metrical data retrieved from the appendicular skeleton and from teeth, in particular the third mandibular molar, are commonly used to evaluate change in animal body size associated with intensive husbandry. Large bodied animals are generally associated with the wild condition. Size diminution over time is thought to reflect the introduction of anthropogenic selective pressures and the onset of domesticated phenotypes with small-bodied animals reflecting their domesticated status^15^. However, especially in the early stages of the domestication process when progenitor species may have been only loosely managed, the relationship between body size and animal ‘domestication’ status is less clear-cut^6^,^7^. Status determination taking into account body size alone can therefore be error-prone. For this study, pig bone specimens were classified as belonging to ‘domesticated, or ‘wild’ according to criteria established independently by numerous zooarchaeologists over the course of several decades. For the majority of pig bone specimens obtained from the northern region, domesticated status was established using pig bone and tooth specimens from the medieval site of Hedeby located in Schleswig-Holstein, northern Germany. For each skeletal element, all Mesolithic or Neolithic bone specimens smaller than Hedeby specimens were classified as ‘domesticated’ (Schmölcke, personal communication). Bone and tooth specimens within the range of modern European wild boar were classified as ‘wild’, while bone and tooth specimens larger than those from Hedeby but smaller than modern wild boar were classified as ‘indeterminate’. The latter were not considered in the present study. Bone specimens recovered from sites located in regions outside of northern Europe were identified as belonging to wild or domesticated animals according to criteria established by individual analysts (Table S1). In addition, this study included mtDNA haplotype information to assist in classification (e.g. specimens yielding Near Eastern Y1 or Y2 lineages were classified as domesticated). In addition, the genetic analysis of a relatively large number of ancient specimens largely allows for zooarchaeological misclassification errors. The type of skeletal element used for aDNA analysis and the archaeological context from which each specimen is derived are presented in Table S1.

### aDNA quality control procedures and mtDNA sequence analysis

All analyses were performed in the aDNA laboratory established at Kiel University, following the strict guidelines and procedures for work with minute amounts of degraded DNA^2^,^16^,^17^. Using previously described methods, mtDNA haplotypes were determined for an 80 bp diagnostic fragment from the control region of the *Sus* mitochondrial genome^2^. All sequencing results are shown in Table S1.

### Stochastic models for the origin of mtDNA haplotypes in northern domestic pigs

In the following, pig subspecies (ps) are denoted by DP (domestic pig) and WB (wild boar), and regional affiliation (r) are denoted by NG (northern group) and SG (southern group). Then, P(h|t_i_, r, ps) is the conditional probability that an animal from subspecies ps living in region r during time period t_i_ carried haplotype h. For the sake of brevity, we define p_NG_(t_i_) as the probability that a northern t_i_ domestic pig descended from a northern t_i−1_ pig (either DP or WB), and p_DP_(t_i_) is the probability that a northern t_i_ domestic pig descended from a domestic t_i−1_ pig, given that this predecessor came from the northern group.

In modelling the origin of mtDNA haplotypes of domestic pigs from the northern group, we will assume that no domestic pigs were present in that group during time period t_1_. A northern t_2_ domestic pig therefore descended either from a northern t_1_ wild boar or from a southern t_1_ domestic pig. This results in the following model equation:


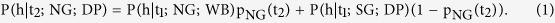


Northern t_3_ domestic pigs are assumed to have descended from (*i*) northern t_2_ domestic pigs, (*ii*) northern t_2_ wild boars or (*iii*) from southern t_2_ domestic pigs. The resulting model equation thus reads


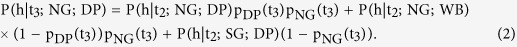


The mathematical models underlying the statistical analyses are relatively simple in several regards. Only one or two parameters are considered and the time and geography are treated as discreet (i.e. three time periods, two geographic regions). These simplifications take into account the limitation of (*i*) the sample size, which prohibits employment of multi-parameter models, and (*ii*) the level of accuracy achievable by way of archaeological dating. Therefore, the models appear adequate for the aims and scope of the present study acknowledging that more refined temporal or geographic structures could not be resolved.

### Parameter estimation

The two model parameters p_NG_(t_i_), the probability that a northern t_i_ domestic pig descended from a northern t_i−1_ pig, and p_DP_(t_i_), the probability that a northern t_i−1_ predecessor was domestic rather than wild, were estimated by maximum likelihood using absolute frequencies n_h_(t_i_), for short n_h_, of haplotypes A, C, Y1 and Y2 in the sampled domestic pigs from the northern group in time period t_i_. Assuming that the sampled pigs were stochastically independent, these data follow a multinomial distribution:


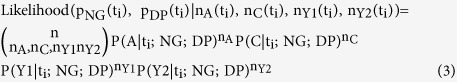


Parameters p_NG_(t_i_) and p_DP_(t_i_) enter into the likelihood model via Equations (1) or (2), depending upon the respective time period. Conditional probabilities P(h|t_1_;SG;DP), P(h|t_2_;NG;DP), P(h|t_2_;NG;WB) and P(h|t_2_;SG;DP) can be estimated from the available mtDNA data. For P(h|t_1_;NG;WB), however, no such data were available so that we had to assume P(h|t_1_;NG;WB) ≈ P(h|t_2_;NG;WB).

Calculations were performed with statistics software R^18^. Numerical maximization of the likelihood was carried out with function optim allowing for box constraints on probabilities^19^. To deal with multiple maxima, we chose starting values from a grid of 12 equally spaced values between 0 and 1 and inspected the likelihood surface by means of a 2D or 3D plot. For the estimation of p_NG_ for time period t_2_, confidence intervals were calculated by bootstrapping (n = 100,000 bootstrap samples). For the estimation of p_NG_ and p_DP_ for time period t_3_, bootstrapping and therefore the calculation of a confidence interval was not feasible because the three-dimensional likelihood surface was too complex.

## Additional Information

**How to cite this article**: Caliebe, A. *et al*. Insights into early pig domestication provided by ancient DNA analysis. *Sci. Rep.* **7**, 44550; doi: 10.1038/srep44550 (2017).

**Publisher's note:** Springer Nature remains neutral with regard to jurisdictional claims in published maps and institutional affiliations.

## Supplementary Material

Supplementary Figure S1

Supplementary Table S1

## Figures and Tables

**Table 1:** mtDNA haplotype distribution in ancient domestic pigs.

time period	northern group					southern group				
	A	C	Y1	Y2	total	A	C	Y1	Y2	total
t_1_ (5500–5000 BC)	*	*	*	*	*	14	8	10	0	32
t_2_ (5000–4000 BC)	9	3	4	0	16	2	2	4	3	11
t_3_ (4000–3000 BC)	11	13	4	0	28	**	**	**	**	**

*Domestic pigs were assumed to lack from the northern group during time period t_1_. **Southern domestic pigs of time period t_3_ are not necessary for stochastic modelling.

**Table 2:** mtDNA haplotype distribution in ancient wild boars.

time period	A	C	Y1	Y2	total
t_2_ (5000–4000 BC)	5	24	0	0	29

**Table 3:** Parameter estimates for the origin of northern domestic pigs.

	2 haplotypes A and C *vs* Y1 and Y2	4 haplotypes A, C, Y1, Y2
t_1_ to t_2_	p_NG_ = 0.20 95% CI: [0, 0.83]	p_NG_ = 0 95% CI: [0, 0.28]
t_2_ to t_3_	0.78 ≤ p_NG_ ≤ 1 0 ≤ p_DP_ ≤ 0.57	p_NG_ = 1 p_DP_ = 0.57

t_1_ to t_2_, origin in time period t_1_ of northern t_2_ domestic pigs; t_2_ to t_3_, origin in time period t_2_ of northern t_3_ domestic pigs; p_NG_, maximum likelihood estimate of the probability that a northern domestic pig descended from a northern pig from the previous time period; p_DP_, maximum likelihood estimate of the probability that a northern predecessor was domestic; see Fig. 3 and S1 for likelihood functions; 95% CI, 95% confidence interval.

## References

[b1] Larson G. . Ancient DNA, pig domestication, and the spread of the Neolithic into Europe. Proc Natl Acad Sci USA 104, 15276–15281 (2007). 17855556 10.1073/pnas.0703411104 PMC1976408

[b2] Krause-Kyora B. . Use of domesticated pigs by Mesolithic hunter-gatherers in northwestern Europe. Nat Commun 4, 2348 (2013). 23982268 10.1038/ncomms3348 PMC3903269

[b3] Vai S. . The Biarzo case in northern Italy: is the temporal dynamic of swine mitochondrial DNA lineages in Europe related to domestication? Sci Rep 5, 16514 (2015). 26549464 10.1038/srep16514 PMC4637886

[b4] Larson G. . Worldwide phylogeography of wild boar reveals multiple centers of pig domestication. Science 307, 1618–1621 (2005). 15761152 10.1126/science.1106927

[b5] Ottoni C. . Pig domestication and human-mediated dispersal in western Eurasia revealed through ancient DNA and geometric morphometrics. Mol Biol Evol 30, 824–832 (2013). 23180578 10.1093/molbev/mss261 PMC3603306

[b6] Rowley-Conwy P. & Zeder M. Mesolithic domestic pigs at Rosenhof – or wild boar? A critical re-appraisal of ancient DNA and geometric morphometrics. World Archaeology 46, 813–824 (2014).

[b7] Rowley-Conwy P. & Zeder M. Wild Boar or Domestic Pigs? Response to Evin *et al*. World Archaeology 46, 835–840 (2014).

[b8] Rowley-Conwy P. Westward Ho! The spread of agriculture from Central Europe to the Atlantic. Curr Anthropol 52, S431–S451 (2011).

[b9] Frantz L. A. . Evidence of long-term gene flow and selection during domestication from analyses of Eurasian wild and domestic pig genomes. Nature genetics 47, 1141–1148 (2015). 26323058 10.1038/ng.3394

[b10] Hübner K.-D., Saur R. & Reichstein H. Palynologische und säugetierkundliche Untersuchungen zum Siedlungsplatz Hüde I am Dümmer Landkreis Diepholz. In Palynologische und säugetierkundliche Untersuchungen zum Siedlungsplatz Hüde I am Dümmer Landkreis Diepholz (eds Jacob-Friesen G., Schütrumpf R., Hübner K.-D., Saur R., Reichstein H.) (Wachholtz 1988).

[b11] Heinrich D. Die Tierknochen des frühneolithischen Wohnplatzes Wangels LA 505. Ein Vorbericht. Offa 54, 43–48 (1998).

[b12] Marshall F. B., Dobney K., Denham T. & Capriles J. M. Evaluating the roles of directed breeding and gene flow in animal domestication. Proc Natl Acad Sci USA 111, 6153–6158 (2014). 24753599 10.1073/pnas.1312984110 PMC4035985

[b13] Scandura M., Iacolina L. & Apollonio M. Genetic diversity in the European wild boar Sus scrofa: phylogeography, population structure and wild x domestic hybridization. Mammal Rev 41, 125–137 (2011).

[b14] White S. From globalized pig breeds to capitalist pigs: a study in animal cultures and evolutionary history. Environ Hist-Us 16, 94–120 (2011).

[b15] Zeder M. A., Emshwiller E., Smith B. D. & Bradley D. G. Documenting domestication: the intersection of genetics and archaeology. Trends in Genetics 22, 139–155 (2006). 16458995 10.1016/j.tig.2006.01.007

[b16] Lee E. . Ancient DNA insights from the Middle Neolithic in Germany. Archaeol Anthrop Sci 6, 199–204 (2014).

[b17] Lee E. J. . Collective burials among agro-pastoral societies in later Neolithic Germany: perspectives from ancient DNA. J Archaeol Sci 51, 174–180 (2014).

[b18] R Core Team. R: A language and environment for statistical computing. R Foundation for Statistical Computing, Vienna, Austria. URL http://www.R-project.org/ (2013).

[b19] Byrd R. H., Lu P. H., Nocedal J. & Zhu C. Y. A limited memory algorithm for bound constrained optimization. Siam J Sci Comput 16, 1190–1208 (1995).

