# Supplementary material for: Insights into early pig domestication provided by ancient DNA analysis
Source: Sci Rep. 2017 Mar 16;7:44550. doi: 10.1038/srep44550 (PMC5353713; doi:10.1038/srep44550)
Supplement: Supplementary Figure S1 [file srep44550-s1.docx]

**Insights into early pig domestication provided by ancient DNA analysis**

Amke Caliebe, Almut Nebel, Cheryl Makarewicz, Michael Krawczak, Ben Krause-Kyora

A

B

**Figure S1, related to Table 1. Likelihood functions for parameter p_NG_ in time period t_2_ (5000-4000 BC).**

p_NG_, probability that a northern t_2_ domestic pig descended from a northern t_1_ wild boar; for the definition of the likelihood function see Experimental Procedures, Section Parameter estimation, Equation (3)$.$

A: likelihood function for two haplotypes (A and C *vs* Y1 and Y2)**;** B: likelihood function for four haplotypes (A,C,Y1,Y2). In A the maximum likelihood estimate of p_NG_ equals 0.20 and in B zero.
